# Strengthening brucellosis prevention and control in Iran: policy priorities informed by stakeholder networks, agenda-setting dynamics, and economic burden evidence

**DOI:** 10.1186/s41182-025-00896-1

**Published:** 2026-01-04

**Authors:** Meysam Behzadifar, Ahad Bakhtiari, Samad Azari, Mariano Martini, Masoud Behzadifar

**Affiliations:** 1 Social Determinants of Health Research Center, Lorestan University of Medical Sciences, Khorramabad, Iran; 2 Health Equity Research Center (HERC), Tehran University of Medical Sciences (TUMS), Tehran, Iran; 3 Health Management and Economics Research Center, Health Management Research Institute, Iran University of Medical Sciences, Tehran, Iran; 4 Department of Health Sciences, University of Genoa, Genoa, Italy

**Keywords:** Brucellosis, One Health, Iran, Health policy, Stakeholder network, Economic burden, Agenda-setting

## Abstract

Brucellosis imposes persistent public health and economic burdens in Iran, yet multisectoral coordination remains limited. Drawing on three published studies, a stakeholder network analysis, a policy agenda-setting assessment, and a cost-of-illness evaluation, this correspondence synthesizes key evidence to identify structural, political, and financial barriers to effective control. Findings reveal fragmented stakeholder engagement, misalignment of problem–policy–politics streams, and a substantial proportion of affected households exposed to catastrophic health expenditure, highlighting major gaps in financial protection. The integrated evidence supports six priority actions, including establishing One Health governance, strengthening vaccination and veterinary capacity, enhancing community engagement, improving financial protection, increasing political prioritization, and expanding international collaboration. These measures are essential for sustainable brucellosis control in Iran.

## Background

Brucellosis is one of the most widespread zoonotic infections globally and remains a major public health challenge, particularly in low- and middle-income countries. The disease affects over half a million people annually and can lead to chronic complications such as osteoarticular disorders, neurobrucellosis, chronic fatigue, and long-term disability, placing substantial pressure on already strained health systems [1].

From an economic perspective, brucellosis contributes to productivity loss, prolonged treatment costs, reduced livestock fertility, decreased milk yield, and high rates of abortion in animals, generating multimillion-dollar losses in agricultural economies [2]. In many endemic regions, persistent transmission is driven by limited access to diagnostic services, incomplete livestock vaccination programs, consumption of unpasteurized dairy products, and insufficient multisectoral coordination across human, animal, and environmental health sectors [3]. These combined health and economic challenges highlight the need for integrated One Health strategies and stronger national policies for effective disease control.

Brucellosis continues to be one of the most significant zoonotic diseases in Iran, particularly in western provinces where livestock production, traditional dairy consumption, and constraints in rural veterinary infrastructure heighten transmission risks [4].

Despite decades of control efforts, incidence remains high, and recurrence patterns persist due to structural, cultural, and financial barriers that impede sustained prevention efforts [5]. Overall, the burden of brucellosis in Iran reflects a combination of long-standing epidemiological challenges, behavioral factors, and gaps in national prevention systems, underscoring the need for renewed policy attention and coordinated multisectoral action.

## Evidence-based and methodological sources

This correspondence synthesizes evidence derived from three previously published studies that examined brucellosis prevention and control in Iran from structural, policy, and economic perspectives [6–8]. These studies provide the empirical foundation for the integrated policy recommendations presented here.

## Stakeholder network analysis (SNA)

A cross-sectional social network analysis was conducted in Lorestan Province to map the interactions, influence, and coordination patterns among key organizations involved in brucellosis prevention [7]. Data were collected from 75 experts representing human health, veterinary, agricultural, environmental, and non-governmental sectors. The study evaluated network density, clustering coefficient, and centrality metrics to identify the structure and strength of multisectoral collaboration**.** This study serves as the primary source of evidence regarding stakeholder coordination challenges and intersectoral engagement gaps [7].

Complementary policy analysis using the multiple-streams framework revealed additional challenges: high disease prevalence, incomplete livestock vaccination, consumption of unpasteurized dairy, unsafe slaughtering practices, weak intersectoral cooperation, and limited public awareness [6]. Critically, while the Ministry of Health and the University of Medical Sciences recognize brucellosis as a priority, other governmental and non-governmental actors show minimal commitment. As a result, the three policy streams—problem, policy, and politics—rarely converge, and a “policy window” for national-level transformation has not fully materialized. Without coordinated political framing and cross-sectoral ownership, prevention programs cannot achieve sustainable compliance or broad population reach [6].

## Policy agenda-setting analysis

A qualitative policy analysis was undertaken using the Multiple Streams Framework (MSF) to examine how brucellosis is framed within Iran’s health and agricultural policy agendas [6]. The study relied on document review and semi-structured interviews with policymakers, health officials, veterinarians, and program managers. It assessed the alignment of the problem**,** policy, and politics streams to identify barriers preventing the issue from advancing on the national agenda. This study identifies gaps related to political prioritization, policy feasibility, and intersectoral responsibility.

## Cost-of-illness (COI) analysis

A cost-of-illness study conducted in western Iran estimated the direct medical, direct non-medical, and indirect costs associated with human brucellosis [8]. The analysis calculated mean total costs per patient, productivity losses, and the proportion of affected households experiencing catastrophic health expenditure (CHE). The study also estimated provincial and national economic burdens. This study provides the economic rationale underlying financial protection and investment-related policy recommendations [8].

Together, these three studies offer complementary methodological perspectives: structural (SNA), political (MSF), and economic (COI) that allow for a multi-dimensional synthesis of evidence. This integrated evidence base supports the identification of feasible, targeted, and multisectoral policy priorities for brucellosis control in Iran.

## Policy priorities

These policy priorities were derived from a synthesis of evidence across the three published studies: stakeholder network analysis, agenda-setting assessment, and economic burden evaluation, and were further refined through expert consultation. Together, these findings inform a set of strategic, multisectoral actions necessary for strengthening brucellosis control in Iran. Importantly, each priority addresses not only what should be done, but also why previous efforts have stalled and what institutional actions are required to move from consensus to implementation.

1. Establish a coordinated One Health governance framework

A unified One Health governance structure is essential to bridging long-standing gaps between human, animal, and environmental health sectors. To date, the absence of a legally mandated coordinating body has resulted in parallel programs, fragmented accountability, and weak enforcement of shared responsibilities. A national One Health steering committee, mandated through interministerial agreement, would institutionalize shared responsibilities for surveillance, data integration, and cross-sectoral decision-making. This committee should have a formal budget line, defined decision-making authority, and performance indicators tied to provincial implementation outcomes. Developing interoperable reporting systems, harmonized outbreak investigation protocols, and joint rapid-response mechanisms would significantly enhance early detection and timely containment of brucellosis outbreaks. Without binding governance arrangements and incentive structures, coordination will remain voluntary and therefore ineffective.

2. Expand community-level engagement and behavioral interventions

Because many transmission pathways arise from deeply rooted cultural and livelihood practices, community-engaged interventions must be tailored to local norms and implemented through trusted social networks. Previous programs have failed largely because risk communication has been centralized, episodic, and disconnected from local authority structures. Engaging NGOs, village councils, cooperative associations, and religious leaders through formal contracts and micro-grant schemes administered by provincial health authorities can increase acceptance of preventive measures and sustain behavior change.

Educational efforts should emphasize the risks associated with unpasteurized dairy consumption, home slaughtering, and informal livestock trade. These messages should be delivered by trained community health volunteers and veterinary outreach workers rather than solely through mass media campaigns. Strengthening social participation is crucial for overcoming resistance to behavioral change and ensuring that prevention strategies are locally owned rather than externally imposed.

3. Improve livestock vaccination coverage and veterinary service accessibility

Increasing livestock vaccination coverage requires reliable national vaccine procurement, enhanced domestic production capacity, and the allocation of targeted subsidies for high-risk rural regions. Historically, inconsistent funding, delayed vaccine distribution, and weak cold-chain infrastructure have undermined vaccination campaigns, particularly in remote provinces. The Iran Veterinary Organization, in collaboration with provincial authorities, should prioritize multi-year vaccine procurement contracts and performance-based financing for high-coverage districts.

Mobile veterinary units, expanded field services, and strengthened cold-chain logistics are necessary to reach underserved communities. Incentive packages, including rural service bonuses and career advancement credits, are required to retain veterinarians in high-incidence areas. Prioritizing mass vaccination in high-incidence provinces is the most direct and cost-effective strategy for interrupting transmission at the animal source.

4. Strengthen financial protection for affected households

Given the high proportion of households experiencing catastrophic health expenditure, financial protection must be integrated as a core component of brucellosis control policy. To date, brucellosis-related services have remained partially excluded from insurance benefit packages, resulting in delayed care-seeking and treatment discontinuation. Health insurance organizations should explicitly include diagnostic testing, prolonged antibiotic regimens, hospital care, and follow-up services for brucellosis within basic coverage schemes.

Livestock compensation mechanisms, financed jointly by agricultural insurance funds and government subsidies, are essential to prevent underreporting and concealment of infected animals. Additionally, targeted subsidies for low-income rural households would mitigate out-of-pocket (OOP) costs and improve compliance with treatment and reporting requirements. Without financial risk protection, public health directives are unlikely to achieve sustained adherence.

5. Mobilize political support by reframing brucellosis as a national economic and food security threat

To achieve durable policy attention, brucellosis must be framed beyond a biomedical issue. One reason for limited political traction has been the perception of brucellosis as a localized rural health problem rather than a national development concern. Evidence from economic burden assessments demonstrates that the disease contributes to productivity loss, household impoverishment, reduced livestock output, and threats to food security.

Policy briefs translating epidemiological and cost-of-illness data into fiscal and agricultural impact scenarios should be prepared jointly by health economists and planning agencies and presented to the Parliament and the Plan and Budget Organization. This reframing is critical for opening a genuine policy window, securing sustained funding, and mobilizing cross-sectoral leadership.

6. Leverage international technical and financial support

Strengthening brucellosis control in Iran would benefit from structured collaboration with the World Health Organization (WHO) and the Food and Agriculture Organization (FAO). Previous engagement has been largely technical and project-based, lacking long-term integration into national systems. Formal cooperation agreements should focus on laboratory accreditation, workforce training, standardized surveillance tools, and evaluation of vaccination strategies.

Participation in regional brucellosis coordination initiatives can enable cross-border data sharing and harmonized control measures, particularly in border provinces. International support should be aligned with national priorities and used strategically to address capacity gaps rather than substitute domestic investment.

Taken together, these policy priorities emphasize that brucellosis control in Iran requires not only technical solutions, but also institutional reform, financial commitment, and political leadership. Clear role allocation, enforceable governance mechanisms, and implementation-focused planning are essential to translating consensus into action.

## Conclusion

Brucellosis continues to impose a significant public health and socioeconomic burden in Iran, particularly in rural and livestock-dependent regions. The synthesis of evidence from stakeholder network analysis, agenda-setting evaluation, and economic burden assessment reveals that persistent transmission is rooted not in biological complexity but in systemic and governance-related shortcomings. Fragmented intersectoral coordination, limited community engagement, and insufficient political prioritization undermine the effectiveness of existing prevention programs. Additionally, the substantial financial burden experienced by affected households highlights the need to integrate economic protection into disease control efforts. A sustainable reduction in brucellosis incidence requires a coordinated national strategy grounded in One Health principles, enabling shared accountability across human, animal, and environmental health sectors. Strengthening veterinary infrastructure, expanding risk communication and behavioral interventions, improving access to livestock vaccination, and enhancing financial protection mechanisms for vulnerable households are essential steps. Equally important is reframing brucellosis as a national development and food security issue to mobilize stronger political commitment and long-term investment. By aligning institutional mandates, improving community participation, and leveraging international expertise, Iran can meaningfully advance toward more effective control of brucellosis. Without decisive action, the human, economic, and social consequences of this preventable disease will continue to intensify.

## Data Availability

Not applicable.

## Abbreviations

CHE: Catastrophic health expenditure
COI: Cost-of-illness
DMC: Direct medical costs
DNMC: Direct non-medical costs
FAO: Food and Agriculture Organization
MSF: Multiple Streams Framework
NGO: Non-governmental organization
OOP: Out-of-pocket
PPP: Purchasing power parity
SNA: Social network analysis
WHO: World Health Organization
